# Identification and Validation of a Five-Gene Signature Associated With Overall Survival in Breast Cancer Patients

**DOI:** 10.3389/fonc.2021.660242

**Published:** 2021-08-26

**Authors:** Xiaolong Wang, Chen Li, Tong Chen, Wenhao Li, Hanwen Zhang, Dong Zhang, Ying Liu, Dianwen Han, Yaming Li, Zheng Li, Dan Luo, Ning Zhang, Qifeng Yang

**Affiliations:** ^1^Department of Breast Surgery, Qilu Hospital of Shandong University, Jinan, China; ^2^Department of Pathology Tissue Bank, Qilu Hospital of Shandong University, Jinan, China; ^3^Research Institute of Breast Cancer, Shandong University, Jinan, China

**Keywords:** breast cancer, bioinformatics, LASSO COX, prognostic biomarkers, risk score, individualized therapy

## Abstract

**Background:**

Recent years, the global prevalence of breast cancer (BC) was still high and the underlying molecular mechanisms remained largely unknown. The investigation of prognosis-related biomarkers had become an urgent demand.

**Results:**

In this study, gene expression profiles and clinical information of breast cancer patients were downloaded from the TCGA database. The differentially expressed genes (DEGs) were estimated by Gene Ontology (GO) analysis and Kyoto Encyclopedia of Genes and Genomes (KEGG) analysis. A risk score formula involving five novel prognostic associated biomarkers (EDN2, CLEC3B, SV2C, WT1, and MUC2) were then constructed by LASSO. The prognostic value of the risk model was further confirmed in the TCGA entire cohort and an independent external validation cohort. To explore the biological functions of the selected genes, *in vitro* assays were performed, indicating that these novel biomarkers could markedly influence breast cancer progression.

**Conclusions:**

We established a predictive five-gene signature, which could be helpful for a personalized management in breast cancer patients.

## Introduction

Breast cancer is the most common diagnosed cancer and the leading cause of cancer-related death in women across the world (1). According to the Cancer Statistics 2020, around 276,480 cases of female breast cancer were diagnosed in the US with the expectation of 42,170 deaths (2). Due to the early detection and the progression in diagnosis and treatments, the mortality rate of breast cancer had declined over the past decades (3). However, for the patients who progressed to metastasis or chemoresistance, the prognosis were still poor (4, 5). Thus, there was an urgent need for the construction of a reliable risk model to evaluate the prognosis of breast cancer patients and identify novel therapeutic targets for individual treatment.

Dysregulation of genes played crucial roles in various biological processes (6). For the limitation on the statistical property of single biomarkers, it was indicated by various studies that multigene signatures provided by systematic analysis could act as more accurate predictive biomarkers than the conventional clinicopathologic characteristics for the risk stratification (7, 8). The 21-gene Oncotype DX Breast Cancer Recurrence Score was developed to evaluate the risk of distant and local recurrence, and estimate the benefit of chemotherapy for the ER-positive breast cancer (9). The MammaPrint 70-gene signature has been proved to improve the prediction of clinical prognosis for early-stage breast cancer patients (10). The EndoPredict testing was composed of a 12-gene molecular score (MS) with the number of positive lymph nodes and tumor size to calculate a single score (EPclin) which was associated with distant recurrence (11). The Prosigna breast cancer assay based on PAM50 (Prediction Analysis of Microarray 50) provided a more valuable prognostic information than the commonly available pathological staging and histological grade (12). Therefore, the identification of novel multigene signatures played a critical role to ameliorate the prognosis of breast cancer patients and provide better treatment strategies for the high-risk population.

Over the past decades, in-depth gene sequencing and bioinformatics provided us the chance to identify novel diagnostic parameters and guide the individual treatment optimization for various illnesses (13–16). Gene expression microarray was an effective method to show large-scale data at genomic levels, and rapid progression of bioinformatics make it possible to mine more reliable biomarkers (17). The Cancer Genome Atlas (TCGA) was an open, public, large-scale database, which contains abundant raw data for cancer researches (18). In our present study, based on the mRNA expression profiles acquired from the TCGA databases, a prognosis‐associated gene signature was constructed by the LASSO Cox regression model (19, 20). Also, the selected biomarkers were proved to play central roles in breast cancer progression by *in vitro* assays.

## Materials and Methods

### Data Source

The mRNA expression profile of breast cancer patients used to identify the differentially expressed genes (DEGs) were derived from the TCGA databases (http://tcga-data.nci.nih.gov/tcga/) on October 1, 2018, which contained 113 normal breast tissues and 1,076 breast tumor tissues. A total of 1,076 patients with clinical information were enrolled in this study. TCGA databases were open-access and publicly available. The present study followed the data access policy and publishing guidelines. In total, 98 patients with pathologically confirmed breast cancer from July 2008 to December 2020 at Qilu Hospital, Shandong University (Jinan, China) were enrolled in the independent external validation cohort. All patients provided a written informed consent before their study entry.

### The Selection of Differentially Expressed Genes

To identify the genes that are differentially expressed in breast cancer tissues and normal tissues, the raw data of mRNA expression were normalized. Gene counts were converted into TPM (transcripts per million mapped reads) values and log2-transformed. R package “limma” was then used to screen the differentially expressed genes (DEGs). The screening conditions for the differentially expressed genes used the following criteria: |fold change (FC)| > 3 and adjusted false-discovery rate (FDR) < 0.01 was applied to find the upregulated and downregulated mRNAs. R package “pheatmap” was used to draw the heatmap.

### Functional Analysis

The Gene Ontology (GO) analysis and Kyoto Encyclopedia of Genes and Genomes (KEGG) analysis were widely used methods for the systematic assessment of biological functional studies on high-throughput genomics data (21–23). In this study, functional enrichment analyses of the GO analysis and KEGG analysis were performed by FunRich, an open access, standalone tool for functional enrichment and network analysis (24). The molecular function, cellular component, biological process, and KEGG pathway of DEGs were estimated. The “ggplot2” package for R software was used to analyze the data.

### Construction of Gene-Related Risk Model for Breast Cancer

The least absolute shrinkage and selection operator (LASSO) method was a commonly used method for regression with high-dimensional predictors (25). In this study, Lasso was used to obtain the most strongly survival-associated genes in the TCGA training cohort. The R packages “survival” and “glmnet” were applied to perform a lasso regression analysis. The mRNA-related gene signature was expressed as follows:

$risk\;score\;=\;\left(coefficient_{gene1\;}\times \;status\;of\;gene\;1\right)\;+\;\left(coefficient_{gene\;2}\times \;status\;of\;gene\;2\right)\;+\;\ldots \;+\;\left(coefficient_{gene\;n}\times status\;of\;gene\;n\right)$ (26).

### Survival Analysis

We analyzed the overall survival of patients by the Kaplan-Meier method. The R package “survival” and “survminer” were applied to construct the Kaplan–Meier survival plots (the difference in survival rates among different groups was measured and p < 0.05 was considered significant in the survival analysis).

### Cell Culture

The human breast cancer cell lines MDA-MB-231 and MDA-MB-468 used in this study were purchased from American Type Culture Collection (ATCC, Manassas, VA, USA), and routinely maintained in the DMEM/high glucose medium (Gibco-BRL, Rockville, IN, USA) with 10% fetal bovine serum (Haoyang Biological Manufacture, Tianjin, China), and 1% penicillin-streptomycin at a 37°C cell culture incubator with 5% CO_2_.

### Transfection and Quantitative Real-Time PCR

Transfection was conducted with Lipofectamine 2000 (Invitrogen) following the protocol of the manufacturer. Generally, 5 × 10^5^ cells were seeded into 6-well plates one day before transfection. When cells reached the 80% confluence, the plasmid DNA and Lipofectamine 2000 were diluted with the Opti-MEM I Reduced Serum Medium, respectively, and then mixed together. After incubating for 20 minutes at room temperature, the mixture was added into the plate drop by drop. After 24–48 h, the cells were used for further experiments.

TRIzol reagent (Invitrogen, Carlsbad, CA, USA) was used to extract RNA from 5 × 10^5^ cells following the protocol of the manufacturer. Total RNA was finally suspended in 20 µL of RNase-free water. The purity and quality of the isolated RNA was evaluated by NanoDrop with A_260_/A_280_ ratio of 1.9–2.0. A total of 500 ng RNA was used to synthesize cDNAs using the PrimeScript reverse transcriptase reagent kit (TaKaRa, Shiga, Japan), according to the protocol of the manufacturer. qRT-PCR was performed with the Roche Light Cycler 480 II using the SYBR Premix Ex Taq I (TaKaRa, Japan). Each reaction contained 2 μL of cDNA in a total volume of 20 μL. Relative RNA abundances were calculated by the standard 2^-ΔΔCt^ method after normalization to GAPDH. The specific primers used in the article are provided in **Supplementary Table 1**.

### 3-(4,5-Dimethyl-2-Thiazolyl)-2,5–Diphenyl-2H-Tetrazolium Bromide Assay

Cell proliferation assay was determined using MTT (Sigma, St. Louis, MO, USA) according to the instructions. MDA-MB-231 and MDA-MB-468 cells were plated into 96-well cell culture plates with at least three replicate wells for each group. Afterwards, 20 μL of MTT (5 mg/mL in PBS) was added to each well and incubated for another 6 h at 37°C. The supernatants were then aspirated carefully and 100 μL of dimethyl sulfoxide (DMSO) was added to each well. Absorbance values were measured using a Microplate Reader (Bio-Rad, Hercules, CA, USA) at 490 nm.

### Colony-Formation Assay

EDN2, CLEC3B, SV2C, and WT1 overexpression cells and control cells were digested by trypsin and seeded in a 6-cm dish at a density of 1,000 cells/dish. MDA-MB-231 cells were cultured for 15 days, MDA-MB-468 cells were cultured for 30 days. Then, the clones were washed by PBS, fixed with methanol for 5 min, and stained with 0.1% crystal violet. Three independent experiments were performed for the same conditions.

### Scratch Assay

After transfected with selected genes, MDA-MB-231 and MDA-MB-468 cells were seeded on a 24-well plate at the density of 1 × 10^5^/well and 1.5 × 10^5^/well, respectively. A straight-line cell-free ‘‘scratch’’ was created by pipette tips and a horizontal line at the back of the plate was drawn as reference point to guarantee the same area of image acquisition. After washing with PBS to remove the debris, the plate was incubated in 5% CO_2_ at 37°C. The migration speed was measured by calculating the difference in the distances between the two edges of the scratch.

### Transwell Assay

Cell migration ability was evaluated by transwell assay using Transwell chamber with a pore size of 8.0 μm (Millipore) according to the instructions of the manufacturer. 1 × 10^5^ MDA-MB-231 cells and 1.5 × 10^5^ MDA-MB-468 cells were suspended in a serum-free medium and plated on upper wells. The medium containing 20% FBS was added to the lower chamber as a chemoattractant. MDA-MB-231 cells were cultured for 12 h, and MDA-MB-468 were cultured for 30 h. After being fixed with methanol for 5 min, the chambers were stained with 1% crystal violet solution for 5 min. Then, the cells in the lower chamber were observed under an inverted microscope. Three independent experiments were performed for the same conditions.

### Western Blotting

The MDA-MB-231 and MDA-MB-468 cells were transfected with EDN2, CLEC3B, SV2C, WT1, and the control cells were transfected with pENTER plasmid. Subsequently, after washing with ice-cold PBS, the proteins of the distinctively treated cells were collected and lysed in a lysis buffer in the presence of protease inhibitors. After centrifugation at 12,000 rpm for 20 min at 4°C, the supernatant was collected. Next, 30 μg of protein were separated by 10% SDS-PAGE and transferred (100 V, 2 h) onto polyvinylidene fluoride (PVDF) membranes (Millipore, Bedford, MA, USA). After blocking with 5% nonfat milk for 1 h, the membranes were incubated overnight at 4°C with the primary antibodies. After washing with TBS-T, the membrane was labeled with the secondary antibody, and protein spots were visualized by ECL. β-actin was used as the endogenous control.

### Statistical Analysis

All the experiments were conducted for the same conditions in triplicate. Statistical analyses in the study were performed with SPSS (version 23.0) and GraphPad Prism 8.0. Kaplan-Meier plots was used to conduct survival analysis. Significant differences were evaluated by student’s t-test and one-way analysis of variance (ANOVA). P-value < 0.05 were considered statistically significant.

## Results

### Clinicopathological Features of Breast Cancer Patients

A total of 1,076 breast cancer patients with clinical information were collected from the TCGA database. By using a random number table, 514 samples were distributed into the TCGA training cohort. Besides, 98 patients diagnosed with breast cancer in Qilu hospital from 2008 to 2020 were enrolled in the Qilu external validation cohort. The detailed demographic and clinicopathological characteristics of the patients involved in the three datasets are shown in **Table 1**.

**Table 1:** Clinical characteristics of patients with breast cancer involved in this study.

	Training set (n = 514)	Entire set (n = 1,076)	External Validation set (n = 98)
Age			
≥65	171 (33.3)	334 (31.0)	11 (11.2)
<65	343 (66.7)	742 (69.0)	87 (88.8)
Sex			
Male	5 (1.0)	12 (1.1)	1 (1.0)
Female	509 (99.0)	1064 (98.9)	97 (99.0)
Primary tumor location			
Left-sided	268 (52.1)	560 (52.0)	53 (54.1)
Right-sided	246 (47.9)	515 (47.9)	45 (45.9)
Unexamined	0 (0)	1 (0.1)	0 (0)
Clinical risk group			
Stage I	86 (16.7)	178 (16.5)	17 (17.3)
Stage II	284 (55.3))	611 (56.8)	49 (50.0)
Stage III	120 (23.3)	247 (22.9)	32 (32.7)
Stage IV	8 (1.6)	20 (1.9)	0 (0)
Unexamined	16 (3.1)	20 (1.9)	0 (0)
T stage			
T1	137 (26.6)	275 (25.6)	37 (37.8)
T2	289 (56.2)	624 (58.0)	53 (54.1)
T3	62 (12.1)	133 (12.4)	1 (1.0)
T4	23 (4.5)	40 (3.7)	7 (7.1)
Unexamined	3 (0.6)	4 (0.3)	0 (0)
N stage			
N0	243 (47.3)	503 (46.7)	36 (36.7)
N1	167 (32.5)	357 (33.2)	32 (32.7)
N2	64 (12.5)	120 (11.1)	17 (17.3)
N3	28 (54.5)	75 (7.0)	13 (13.3)
Unexamined	12 (2.3)	21 (2.0)	0 (0)
M stage			
M0	423 (82.3)	898 (83.5)	98 (100.0)
M1	10 (1.9)	22 (2.0)	0 (0)
Unexamined	81 (15.8)	156 (14.5)	0 (0)
ER status			
ER positive	377 (73.3)	792 (73.6)	61 (62.3)
ER negative	113 (22.0)	233 (21.7)	35 (35.7)
Unexamined	24 (4.7)	51 (4.7)	2 (2.0)
PR status			
PR positive	317 (61.7)	684 (63.6)	60 (61.2)
PR negative	170 (33.1)	338 (31.4)	37 (37.8)
Unexamined	27 (5.2)	54 (5.0)	1 (1.0)
Her-2 status			
Her-2 positive	92 (17.9)	192 (17.8)	26 (26.5)
Her-2 negative	344 (66.9)	739 (68.7)	64 (65.3)
Unexamined	78 (15.2)	145 (13.5)	8 (8.2)
Margin status			
Margin positive	32 (6.2)	78 (7.2)	1 (1.0)
Margin negative	434 (84.4)	904 (84.0)	97 (99.0)
Close	13 (2.5)	26 (2.4)	0 (0)
Unexamined	35 (6.9)	68 (6.4)	0 (0)
Recurrence			
Yes	28 (5.4)	65 (6.0)	33 (33.7)
No	251 (48.8)	508 (47.2)	47 (48.0)
Unexamined	235 (45.8)	503 (46.8)	18 (18.4)
Period of follow up			
Years 0-1	193 (37.5)	399 (37.1)	7 (7.1)
Years 2-4	108 (21.0)	228 (21.2)	16 (16.3)
Years 5-9	41 (8.0)	90 (8.4)	67 (68.4)
Years≥10	12 (2.3)	26 (2.4)	8 (8.2)
Unexamined	160 (31.0)	333 (30.9)	0 (0)
Death of disease			
Yes	48 (9.3)	93 (8.6)	27 (27.6)
No	306 (59.5)	650 (60.4)	71 (72.4)
Unexamined	160 (31.1)	333 (31.0)	0 (0)

Data are n (%).

In the TCGA training cohort, the median age was 58 years (range, 27–90 years). The percentages of patients at clinical stages I, II, III, and IV were 16.7%, 55.3%, 23.3%, and 1.6%, respectively. 71.2% of the patients received mastectomy, 47.3% underwent chemotherapy, 49.0% received radiotherapy, and 26.3% were treated with hormonal therapy. The follow-up periods encompassed the different pathological stages of breast cancer. The median follow-up periods were 592 days (range, 2–6,434 days). A total of 354 patients had prognosis information. During the follow-up, 48/354 (13.6%) of the patients died.

In the TCGA entire cohort, the median age was 58 years (range, 26–90 years). The percentages of patients at clinical stages I, II, III, and IV in the TCGA entire cohort were 16.5%, 56.8%, 22.9%, and 1.9%, respectively. 69.8% of the patients received mastectomy, 48.4% underwent chemotherapy, 50.4% received radiotherapy, and 27% were treated with hormonal therapy. A total of 743 patients had prognosis information. The median follow-up periods were 608 days (range, 1–7,125 days). During the follow-up, 93/743 (12.5%) of the patients died.

As for the Qilu external validation cohort, the median age was 48.5 years (range, 26–81 years). The percentages of clinical stages I, II, III, and IV were 17.3%, 50.0%, 32.7%, and 0%, respectively. In addition, the percentages of histological grades I, II, and III were 2%, 63.3%, and 34.7%, respectively. 96.9% of the patients in the cohort received mastectomy, 92.9% underwent chemotherapy, 37.8% received radiotherapy, and 56.1% were treated with hormonal therapy. The median follow-up periods were 2,841 days (range, 123–4,139 days). A total of 27 out of 98 (27.6%) of the patients died during the follow-up.

### Identification of Differentially Expressed Genes

**Figure 1:**
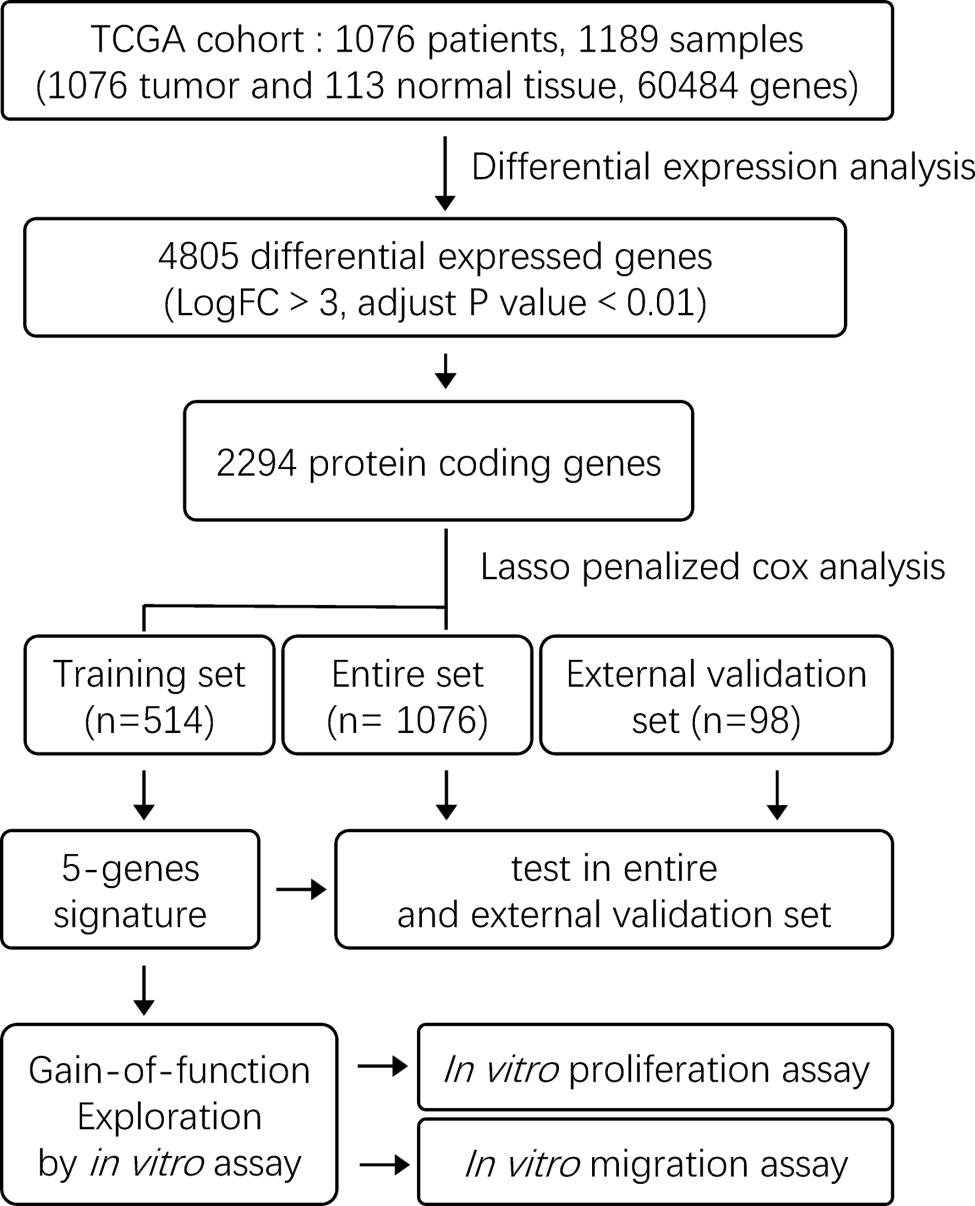
The flow chart showing the scheme of the study on five-gene prognostic signatures for breast cancer.

**Figure 2:**
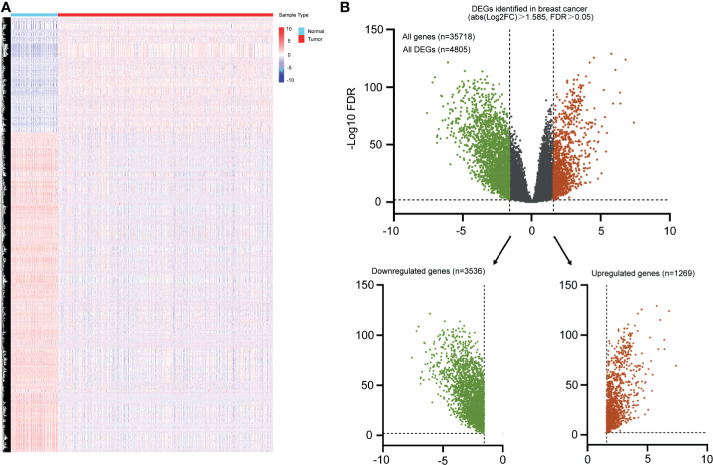
Heatmap and volcano plot were used to show the DEGs in breast cancer. **(A)** Heatmap represented mRNAs differentially expressed between breast cancer and normal breast tissues based on microarray analysis. **(B)** Volcano plot represented all differential expressed genes, green indicated downregulated genes, and red indicated all upregulated genes.

The exploration process of this study is shown in **Figure 1**. Firstly, the differentially expressed genes were initially screened between normal and tumor tissues. Thresholds were set as fold change > 3 and FDR < 0.01 (**Figure 2A**). Volcano plots were used to show the differentially expressed genes (**Figure 2B**). A total of 4,805 genes had differential expressions between normal and tumor tissues, which consisted of 1,269 upregulated genes and 3,536 downregulated genes. In total, 2,294 DEGs were with protein coding functions.

### Enrichment Analyses of Differentially Expressed Genes

To further understand the function of the DEGs, the differentially expressed mRNAs were incorporated into functional annotation analyses. The molecular processes during the progression of breast cancer were investigated through GO enrichment analyses and KEGG pathway analyses.

**Figure 3:**
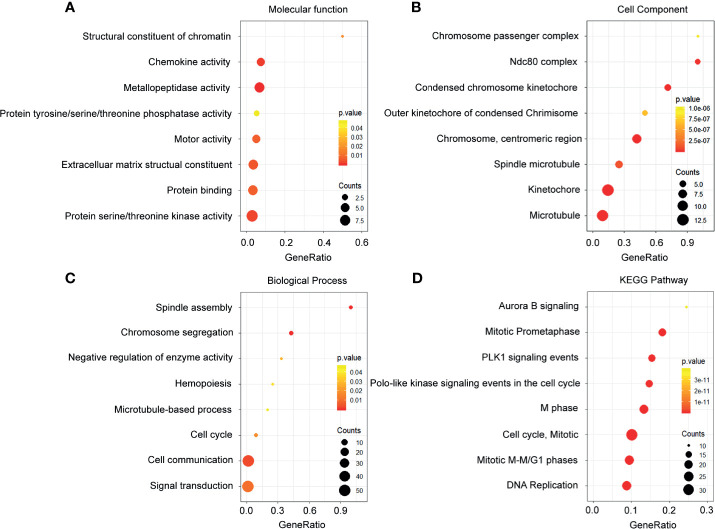
Functional enrichment analysis of the upregulated genes. **(A)** Enrichment of molecular function. **(B)** Enrichment of cellular component. **(C)** Enrichment of biological process. **(D)** Enrichment of Kyoto Encyclopedia of Genes and Genomes.

The upregulated mRNAs associated with molecular function were enriched in the modulation of the structural constituent of chromatin, chemokine activity, and metallopeptidase activity (**Figure 3A**). In terms of the cellular component, the upregulated genes were tightly corresponding to the chromosome passenger complex, Ndc80 complex, and condensed chromosome kinetochore (**Figure 3B**). Additionally, in the analysis on the biological process, spindle assembly, chromosome segregation, and negative regulation of enzyme activity were the most enriched terms mediated by the upregulated genes (**Figure 3C**). From the prospective of the KEGG pathways, the high expression genes were closely related to aurora B signaling, mitotic prometaphase, and PLK1 signaling events (**Figure 3D**)

**Figure 4:**
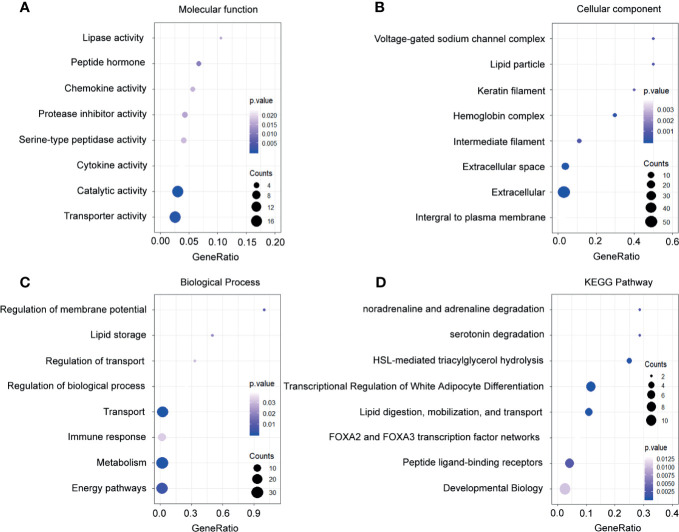
Functional enrichment analysis of the downregulated genes. **(A)** Enrichment of molecular function. **(B)** Enrichment of cellular component. **(C)** Enrichment of biological process. **(D)** Enrichment of Kyoto Encyclopedia of Genes and Genomes.

Meanwhile, the downregulated genes related to molecular function were enriched in the lipase activity, serotonin degradation, and chemokine activity (**Figure 4A**). Through the investigation on the cellular component, the most enriched terms were voltage-gated sodium channel complex, lipid particle, and keratin filament (**Figure 4B**). In the exploration of the biological process, the downregulated genes in breast cancer were enriched in the regulation of membrane potential, lipid storage, and regulation of transport (**Figure 4C**). Besides, analysis on the KEGG pathways proved that the downregulated genes were associated with noradrenaline and adrenaline degradation, serotonin degradation, and HSL-mediated triacylglycerol hydrolysis (**Figure 4D**).

### Construction and Validation of the Risk Prognostic Scoring System in the The Cancer Genome Atlas Training Set

**Figure 5:**
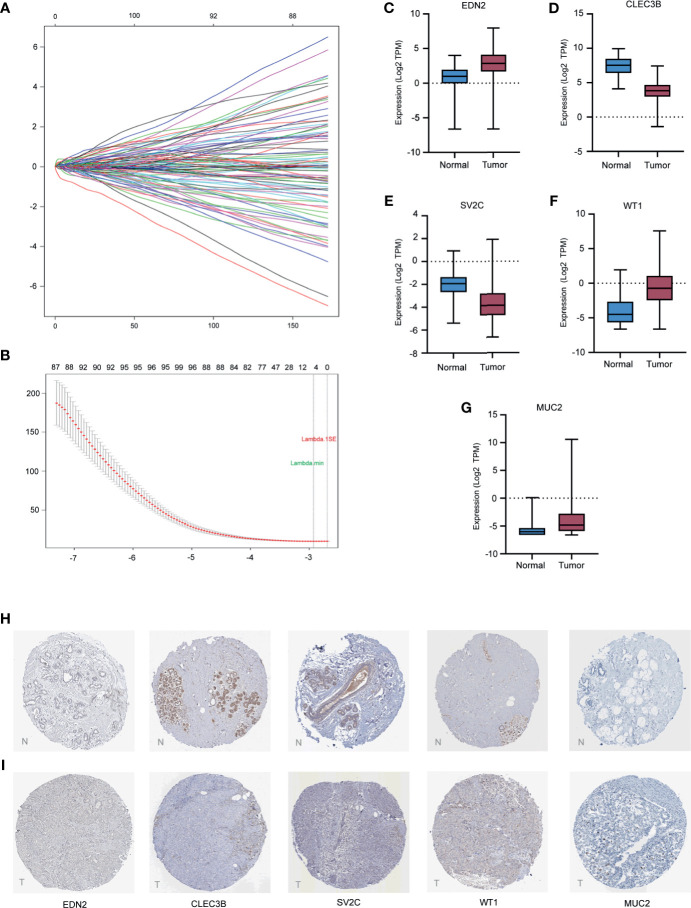
Construction of the five-gene prognostic model and validation of expression of the five genes in breast cancer. **(A)** the coefficients of variables identified based on the LASSO Cox regression model. **(B)** 10-fold Cross validation of LASSO regression. Left and right vertical dotted lines represented the “lambda.min” and “lambda.1se” criteria, respectively. The red dots indicated partial likelihood deviance values, and the gray lines indicated the corresponding standard error. **(C–G)** The mRNA expression levels of selected genes in the TCGA training cohort. **(H, I)** The representative protein expression of the five genes in breast cancer tumor tissue and normal tissue. Data were obtained from the human protein atlas.

A total of 2,294 protein coding genes were further selected using LASSO regression analysis, and cross validation was used to select the penalty parameters (**Figure 5A**). Five genes were identified as the risk factors with LASSO Cox regression analysis and “lambda.min” parameters (**Figure 5B**). The genes obtained in the steps above were inserted into a formula. The expression statuses of the five independent prognostic factors and their correlation coefficients in the LASSO regression model were then used to construct prognostic signatures. Detailed information and the significance of survival prediction by the five genes are presented in **Table 2**.

**Table 2:** Five genes in the signature identified by the LASSO Cox regression analysis.

Gene symbol	Full name	Coefficient
EDN2^1^	Endothelin 2	0.014
CLEC3B^2^	C-type lectin domain family 3 member B	-0.196
SV2C	Synaptic vesicle glycoprotein 2C	0.227
WT1^3^	WT1 transcription factor	0.075
MUC2^4^	Mucin 2, oligomeric mucus/gel-forming	0.113

^1^ET-2, ET2, PPET2. ^2^TN, TNA. ^3^AWT1, GUD, NPHS4, WAGR, WIT-2, WT33. ^4^MLP, MUC-2, SMUC.

Risk score = (expression status of EDN2 × 0.014) + (expression status of CLEC3B × −0.196) + (expression status of SV2C × 0.227) + (expression status of WT1 × 0.075) + (expression status of MUC2 × 0.113)

Of the five biomarkers, three genes (EDN2, WT1, and MUC2) were upregulated in the breast cancer samples while CLEC3B and SV2C were decreased (**Figures 5C–G**). Moreover, the protein expression of the five genes were further explored in the Human Protein Atlas (HPA) and their representative pictures are shown in **Figures 5H, I**.

**Figure 6:**
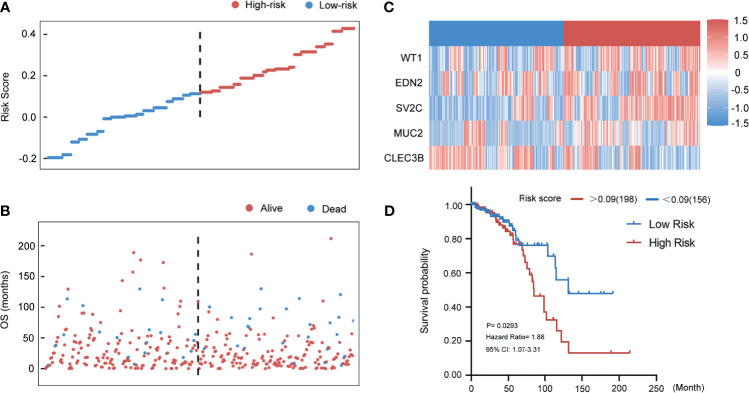
Evaluating the predictive power of five-gene signature in the training group. **(A)** Distribution of risk score. **(B)** Survival status of breast cancer patients in the training group. **(C)** Heatmap of the prognosis-associated gene expression profiles in the TCGA training cohort. **(D)** Kaplan‐Meier plot of the high‐ and low‐risk groups in the training group.

In the TCGA training cohort, the distributions of the risk score of breast cancer patients and the relationships between risk score and survival time are visualized in **Figures 6A, B**. The mRNA expression levels of the selected genes of the patients are shown in **Figure 6C**. Patients in the TCGA training cohort were then assigned to a high- or low-risk score group using the cut-off value (0.09) obtained with the “survival” and “survminer” packages. A total of 198 (56%) patients in the TCGA training cohort were categorized to the high-risk group (RS > 0.09) and 156 (44%) to the low-risk group (RS ≤ 0.09). High-risk patients also had a markedly shorter OS (HR 1.88, 95% CI 1.07–3.31, p < 0.05) *vs*. low-risk patients in the TCGA training cohort (**Figure 6D**).

### Validation of the Five Genes-Model in the TCGA Entire Set

**Figure 7:**
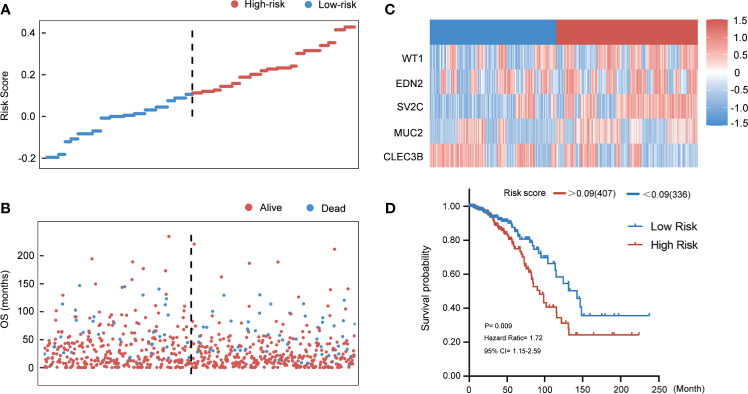
Evaluating the predictive power of five-gene signature in the entire group. **(A)** Distribution of risk score. **(B)** Survival status of breast cancer patients in the entire group. **(C)** Heatmap of the prognosis-associated gene expression profiles in the TCGA entire cohort. **(D)** Kaplan‐Meier plot of the high‐ and low‐risk groups in the entire group.

To assess the stability and reliability of the five genes signature, the result was also tested in the TCGA entire cohort. According to the same risk score that was acquired from the training group, 389 breast cancer patients with follow-up information were divided into high- and low-risk groups. **Figures 7A, B** show the distributions of the risk score of breast cancer patients and the relationships between risk score and survival time. Expressions of the five genes in the risk score formula in the entire group are provided in **Figure 7C**. The relationship between the distribution of risk score and clinical information indicated that the higher patients ranking predicted a poorer overall survival (HR 1.72, 95% CI 1.15–2.59, p < 0.01) (**Figure 7D**).

### Validation of the Five Genes-Model in the Qilu External Validation Set

**Figure 8:**
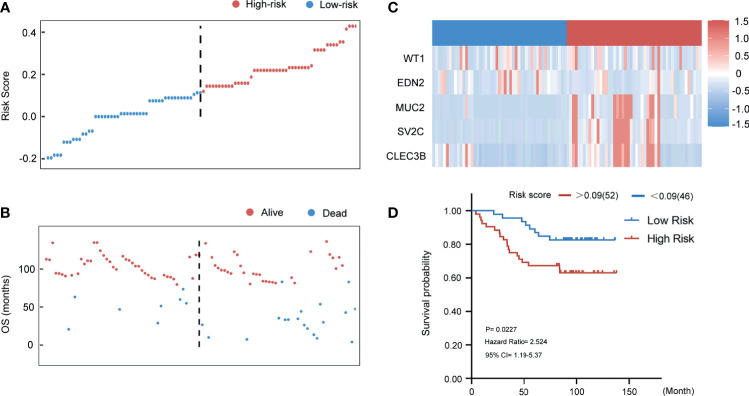
Evaluating the predictive power of five-gene signature in the external validation group. **(A)** Distribution of risk score. **(B)** Survival status of breast cancer patients in the external validation group. **(C)** Heatmap of the prognosis-associated gene expression profiles in the Qilu external validation cohort. **(D)** Kaplan‐Meier plot of the high‐ and low‐risk groups in the external validation group.

In **Figures 8A, B**, patients in the Qilu external validation cohort (n = 98) were divided into the high‐risk group and the low‐risk group according to the same cut-off value of 0.09. The risk score distributions and the survival status were exhibited. The expressions of the five selected genes in 98 patients are also shown in **Figure 8C**. Thus, patients in the Qilu external validation cohort were then categorized to the high-risk group (n = 52) and low-risk group (n = 46). The results of survival analysis were showed in the Kaplan-Meier plot (**Figure 8D**). With the extension of the survival time, the survival rate of the high-risk group became lower, and had a poor prognosis effect (HR 2.524, 95% CI 1.19–5.37, p < 0.05).

### Gain-of-Function Assay of Selected Genes

**Figure 9:**
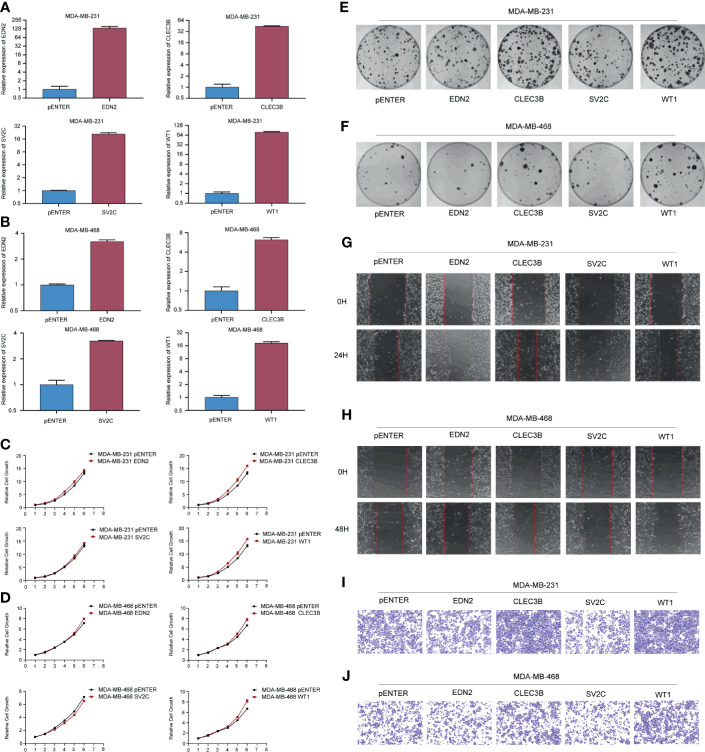
Gain-of-function assay of selected genes regulating cell proliferation and metastasis. **(A, B)** The overexpression efficiency of the selected genes in MDA-MB-231 and MDA-MB-468 cells. **(C, D)** Effect of the selected genes on cell proliferation was tested by MTT in MDA-MB-231 and MDA-MB-468 cells. **(E, F)** Effect of the selected genes on cell proliferation was tested by colony formation assay in MDA-MB-231 and MDA-MB-468 cells. **(G, H)** Effect of the selected genes on cell migration was tested by scratch assay in MDA-MB-231 and MDA-MB-468 cells. **(I, J)** Effect of the selected genes on cell migration was tested by transwell assay in MDA-MB-231 and MDA-MB-468 cells.

As shown in **Supplementary Figure 1**, we have examined the predictive values of EDN2, CLEC3B, SV2C, WT1, and MUC2 on the overall survival in TCGA and METABRIC. According to the results, EDN2, CLEC3B, SV2C, and WT1 could significantly influence the prognosis of breast cancer patients. However, MUC2 was not associated with prognosis. Therefore, we transfected cells with EDN2, CLEC3B, SV2C, and WT1 to investigate the biological functions of these prognosis associated genes. The overexpression efficiency of the selected genes was verified by qRT-PCR (**Figures 9A, B** **)**. We tested the cell proliferative viability using the MTT assay in MDA-MB-231 and MDA-MB-468 cells. As shown in **Figures 9C, D**, overexpression of CLEC3B and WT1 could significantly promote the growth in both cell lines, while EDN2 and SV2C had no obvious influence on cell proliferation. The results were then further validated by the clone formation assay (**Figures 9E, F** **)**. Overexpression of CLEC3B and WT1 could dramatically promote the formation of colonies in breast cancer cells.

Furthermore, cell migration assays were used to evaluate the regulative effects of the selected genes on cell migration. As evidenced by the scratch assay and transwell assay, the mobility of MDA-MB-231 and MDA-MB-468 cells overexpressed CLEC3B and WT1 were considerably increased compared with the control group (**Figures 9G–J**). On the contrary, transfected with SV2C could significantly inhibit cell migration in both breast cancer cell lines, while the function of EDN2 was slight.

### Influence of Selected Genes on the Epithelial-Mesenchymal Transition Signaling Pathway in Breast Cancer

**Figure 10:**
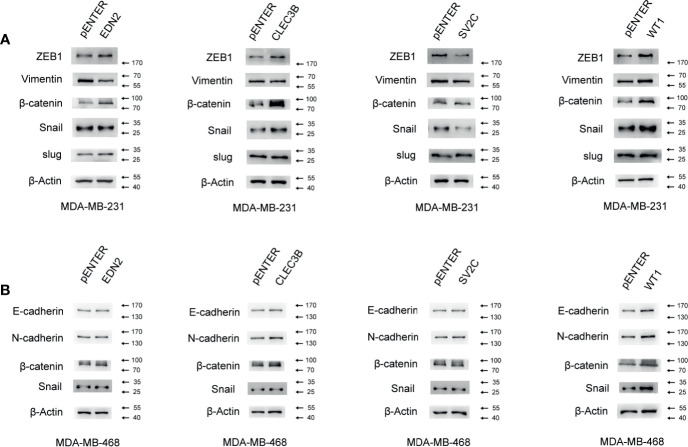
Influence of the selected genes on the EMT signaling pathway in breast cancer. **(A)** Effect of the selected genes on the protein level of the EMT signaling pathway was measured by Western blot assay in MDA-MB-231. **(B)** Effect of the selected genes on the protein level of the EMT signaling pathway was measured by Western blot assay in MDA-MB-468.

The EMT represented a biological process during which polarized epithelial cells lost their cell identity and experienced various biochemical alterations that allowed it to assume mesenchymal phenotypes (27). Normally observed during embryonic development, EMT could also be involved in various pathological conditions. Once hijacked by cancer cells, EMT often led to an enhanced migration capability, acquisition of resistance to apoptosis, and increased cell proliferation (27–30). Thus, we examined the role of the selected genes in the EMT signaling pathway in breast cancer cells. As shown in **Figure 9**, the gain-of-function of EDN2, CLEC3B, and WT1 markedly increased the ZEB1 and β-catenin in MDA-MB-231. Besides, CLEC3B and WT1 could also enhance the expression of snail (**Figure 10A**). By contrast, SV2C seemed to play a key role as a tumor suppressor in the EMT signaling pathway. MDA-MB-231 cells transfected with SV2C showed a low expression of EMT markers, such as ZEB1, vimentin, β-catenin, and snail. In MDA-MB-468, EDN2, CLEC3B, and WT1 were proved to be able to upregulate the protein level of β-catenin (**Figure 10B**). CLEC3B and WT1 transfection led to a higher expression level of N-Cadherin. Moreover, a markedly increase of snail was also observed in the WT1 overexpressed MDA-MB-468 cells.

## Discussion

As one of the most malignant tumors in women, breast cancer was a heterogeneous disease with diverse subtypes. Each subtype had distant biological and clinical characteristics (31). It was of great importance to investigate the underlying molecular pathogenesis of breast cancer and find reliable prognostic biomarkers for the identification of patients with high risk (32). Microarray data had been proved as an effective tool in the identification of gene biomarkers, which was a crucial step for tumor assessment (33). In the present study, gene expression profiles of breast cancer samples and corresponding normal tissue were download from TCGA together with the clinical information. An independent validation cohort was also employed to ensure the stability of the prognostic model. Candidate genes were prescreened by the analysis of the differentially expressed genes between breast cancer and control samples.

In total, 4,805 DEGs were identified. To further investigate the molecular mechanisms involved in breast cancer, GO and KEGG analysis were performed (21, 34). As shown in our data, the upregulated DEGs were mainly enriched in the DNA repair machinery, enhancing cell mobility and limitless replicative potential. PLK1 was a serine/threonine protein kinase, which played a critical role in the regulation of cell cycle and chemoresistance (35, 36). Our data demonstrated that the upregulated DEGs were highly associated with the PLK1 signaling pathway. This indicated that the dysregulation of the PLK1 signaling pathway contributed to the prognosis of breast cancer patients. As for the downregulated DEGs, the enriched terms included correspond to immune response, chemokine activity, and regulation of metabolism. These findings were also consistent with previous breast cancer studies (37–39).

Penalized methods had aroused much attention as a novel predicting tool for high accuracy and good feasibility (40). L_1_-penalty, also known as LASSO, was the most widely used penalty in a high-dimensional cancer classification (25). Recent studies had showed that LASSO could be used as an effective tool in the exploration of potential biomarkers in breast cancer. A 6-KIFs-based risk score (KIF10, KIF15, KIF18A, KIF18B, KIF20A, KIF4A) reported by Li et al. (41) was proved to be associated with the prognosis in patients with breast cancer. Immune-related index in breast cancer were also found through LASSO by Xie et al. (42) and Zheng et al. (43). These researchers indicate that gene signatures could serve as risk factors for cancer management and play a vital role in predicting cancer prognosis.

In our study, to further explore the prognosis-related biomarkers in breast cancer, LASSO Cox regression model was performed. We screened out five protein coding genes (EDN2, CLEC3B, SV2C, WT1, and MUC2) significantly corresponding to the overall survival time of patients with breast cancer in the training group. Compared to a single biomarker alone, the risk score consisted of the coefficient, and expression status of multiple genes markedly increased the reliability and accuracy of diagnosis result. Thus, a 5-genes signature was established as potential biological indicators for breast cancer diagnosis and prognosis. The gene signature was also tested in the TCGA entire cohort and the Qilu external validation cohort. The Kaplan-Meier plot showed the significant difference of the overall survival between the high- and low-risk groups. The 5-gene prognostic model was expected to work as an auxiliary predicting tool in the individual management of breast cancer.

Through the literature search, it was found that several biomarkers related to the gene signature were reported to be involved in the process of cancer development and progression. EDN2 had been reported to be an oncogene overexpressed in various malignancies, which corelated to cell differentiation, proliferation, migration, and resistance to chemotherapy (44–48). However, functions of EDN2 in breast cancer had not been reported. Besides, CLEC3B seemed to play distinct roles in different human cancers. While functioning as tumor suppresser in lung cancer (49), expression of CLEC3B was proved to be related to a poor prognosis in colorectal cancer and gastric cancer (50, 51). WT1 was firstly identified as a tumor suppressor gene in nephroblastoma (52). However, it was demonstrated by subsequent studies that WT1 was related to the disruption of the EMT signaling pathway and docetaxel resistance in breast cancer, high expression of WT1 also corresponded to a lower overall survival (52, 53). Functioned as an oncogene, MUC2 was highly expressed in mucin secreting breast cancers and played a pivotal role in regulating cell proliferation, metastasis, and apoptosis (54).

To further validate the functions of the biomarkers, *in vitro* assays were then performed to evaluate the influence of the selected genes on proliferation and metastasis. We proved that EDN2 could be associated with the protein level in the EMT signaling pathway. It was found that CLEC3B and WT1 could markedly enhance the capability of growth and migration in breast cancer cell lines. Meanwhile, overexpressing SV2C could decrease the cell mobility *in vitro*. Detection of the protein levels further proved that the change in migration ability was probably caused by the alteration of the EMT signaling pathway. In the present study, we established a novel five-gene signature which was a promising tool in predicting breast cancer prognosis. Three of the genes in the five-gene signature were reported to be related to breast cancer for the first time. These potential biomarkers could be helpful for future investigation.

However, there were some limitations which need to be mentioned in this study. First, only the overall survival was taken into consideration, and the quality of life of breast cancer patients were not covered. Besides, for the present research was retrospective, the predicting model should also be testified in the large-scale prospective studies. Thus, the results demanded to be further verified before its application into clinical practice.

## Conclusion

In summary, a five-gene (EDN2, CLEC3B, SV2C, WT1, and MUC2) based prognostic model was constructed and validated in the study, which was proved to be an accurate classifier for risk stratification and clinical decision-making. This study provided us a new angle to better understand the molecular network in malignancies. These selected genes tightly corresponded to the prognosis of breast cancer and might serve as potential biomarkers for future individual treatment.

## Data Availability Statement

The datasets presented in this study can be found in online repositories. The names of the repository/repositories and accession number(s) can be found in the article/**Supplementary Material**.

## Ethics Statement

The studies involving human participants were reviewed and approved by the Ethics Committee on Scientific Research of Shandong University, Qilu Hospital. The patients/participants provided their written informed consent to participate in this study.

## Author Contributions

Conceptualization: QY and XW. Data curation: CL, TC, HZ, YLiu, and DH. Investigation: CL, DL, and NZ. Methodology: XW and WL. Original Draft Preparation: CL. Review and Editing: XW. Figure Preparation and Editing: CL, YLi, ZL, DZ, and XW. Supervision: QY. Project Administration: QY. Funding Acquisition: QY. All authors contributed to the article and approved the submitted version.

## Funding

This work was supported by the National Natural Science Foundation of China (No. 81672613; No. 81874119; No. 82072912; No. 82004122), Shandong Provincial Natural Science Foundation, China (No. ZR2019LZL003, No. ZR201911010260, No. ZR201911050391), Special Foundation for Taishan Scholars (No. ts20190971), Special Support Plan for National High Level Talents (Ten Thousand Talents Program W01020103), National Key Research and Development Program (No. 2018YFC0114705), Funded by Clinical Research Center of Shandong University (No.2020SDUCRCA015), and Qilu Hospital Clinical New Technology Developing Foundation (No. 2018-7; No. 2019-3).

## Conflict of Interest

The authors declare that the research was conducted in the absence of any commercial or financial relationships that could be construed as a potential conflict of interest.

## Publisher’s Note

All claims expressed in this article are solely those of the authors and do not necessarily represent those of their affiliated organizations, or those of the publisher, the editors and the reviewers. Any product that may be evaluated in this article, or claim that may be made by its manufacturer, is not guaranteed or endorsed by the publisher.

## Abbreviations

TCGA, The Cancer Genome Atlas; LASSO, the least absolute shrinkage and selection operator; DEGs, the differentially expressed genes; BC, breast cancer; GO, Gene Ontology; KEGG, the Kyoto Encyclopedia of Genes and Genomes; qRT-PCR, Quantitative Real-Time PCR; MTT, 3-(4,5-dimethyl-2-thiazolyl)-2,5–diphenyl-2H-tetrazolium Bromide; OS, overall survival; RS, risk score; HR, hazard ratio.
