# Supplementary material for: Identification and Validation of a Five-Gene Signature Associated With Overall Survival in Breast Cancer Patients
Source: Front Oncol. 2021 Aug 26;11:660242. doi: 10.3389/fonc.2021.660242 (PMC8428534; doi:10.3389/fonc.2021.660242)
Supplement: Supplementary file 3 [file Table_1.docx]

| **Clinical characteristics of patients with breast cancer involved in this study** | | | |
| --- | --- | --- | --- |
|  | Training set  (n=514) | Entire set  (n=1076) | External Validation set (n=98) |
| Age | | | |
| ≥65 | 171 (33.3) | 334 (31.0) | 11 (11.2) |
| ＜65 | 343 (66.7) | 742 (69.0) | 87 (88.8) |
| Sex | | | |
| Male | 5 (1.0) | 12 (1.1) | 1 (1.0) |
| Female | 509 (99.0) | 1064 (98.9) | 97 (99.0) |
| Primary tumor location | | | |
| Left-sided | 268 (52.1) | 560 (52.0) | 53 (54.1) |
| Right-sided | 246 (47.9) | 515 (47.9) | 45 (45.9) |
| Unexamined | 0 (0) | 1 (0.1) | 0 (0) |
| Clinical risk group | | | |
| Stage I | 86 (16.7) | 178 (16.5) | 17 (17.3) |
| Stage II | 284 (55.3)) | 611 (56.8) | 49 (50.0) |
| Stage III | 120 (23.3) | 247 (22.9) | 32 (32.7) |
| Stage IV | 8 (1.6) | 20 (1.9) | 0 (0) |
| Unexamined | 16 (3.1) | 20 (1.9) | 0 (0) |
| T stage | | | |
| T1 | 137 (26.6) | 275 (25.6) | 37 (37.8) |
| T2 | 289 (56.2) | 624 (58.0) | 53 (54.1) |
| T3 | 62 (12.1) | 133 (12.4) | 1 (1.0) |
| T4 | 23 (4.5) | 40 (3.7) | 7 (7.1) |
| Unexamined | 3 (0.6) | 4 (0.3) | 0 (0) |
| N stage | | | |
| N0 | 243 (47.3) | 503 (46.7) | 36 (36.7) |
| N1 | 167 (32.5) | 357 (33.2) | 32 (32.7) |
| N2 | 64 (12.5) | 120 (11.1) | 17 (17.3) |
| N3 | 28 (54.5) | 75 (7.0) | 13 (13.3) |
| Unexamined | 12 (2.3) | 21 (2.0) | 0 (0) |
| M stage | | | |
| M0 | 423 (82.3) | 898 (83.5) | 98 (100.0) |
| M1 | 10 (1.9) | 22 (2.0) | 0 (0) |
| Unexamined | 81 (15.8) | 156 (14.5) | 0 (0) |
| ER status | | | |
| ER positive | 377 (73.3) | 792 (73.6) | 61 (62.3) |
| ER negative | 113 (22.0) | 233 (21.7) | 35 (35.7) |
| Unexamined | 24 (4.7) | 51 (4.7) | 2 (2.0) |
| PR status | | | |
| PR positive | 317 (61.7) | 684 (63.6) | 60 (61.2) |
| PR negative | 170 (33.1) | 338 (31.4) | 37 (37.8) |
| Unexamined | 27 (5.2) | 54 (5.0) | 1 (1.0) |
| Her-2 status | | | |
| Her-2 positive | 92 (17.9) | 192 (17.8) | 26 (26.5) |
| Her-2 negative | 344 (66.9) | 739 (68.7) | 64 (65.3) |
| Unexamined | 78 (15.2) | 145 (13.5) | 8 (8.2) |
| Margin status | | | |
| Margin positive | 32 (6.2) | 78 (7.2) | 1 (1.0) |
| Margin negative | 434 (84.4) | 904 (84.0) | 97 (99.0) |
| Close | 13 (2.5) | 26 (2.4) | 0 (0) |
| Unexamined | 35 (6.9) | 68 (6.4) | 0 (0) |
| Recurrence | | | |
| Yes | 28 (5.4) | 65 (6.0) | 33 (33.7) |
| No | 251 (48.8) | 508 (47.2) | 47 (48.0) |
| Unexamined | 235 (45.8) | 503 (46.8) | 18 (18.4) |
| Period of follow up | | | |
| Years 0-1 | 193 (37.5) | 399 (37.1) | 7 (7.1) |
| Years 2-4 | 108 (21.0) | 228 (21.2) | 16 (16.3) |
| Years 5-9 | 41 (8.0) | 90 (8.4) | 67 (68.4) |
| Years≥10 | 12 (2.3) | 26 (2.4) | 8 (8.2) |
| Unexamined | 160 (31.0) | 333 (30.9) | 0 (0) |
| Death of disease | | | |
| Yes | 48 (9.3) | 93 (8.6) | 27 (27.6) |
| No | 306 (59.5) | 650 (60.4) | 71 (72.4) |
| Unexamined | 160 (31.1) | 333 (31.0) | 0 (0) |

Data are n (%).
