# Supplementary material for: Identification and Validation of a Five-Gene Signature Associated With Overall Survival in Breast Cancer Patients
Source: Front Oncol. 2021 Aug 26;11:660242. doi: 10.3389/fonc.2021.660242 (PMC8428534; doi:10.3389/fonc.2021.660242)
Supplement: Supplementary file 4 [file Table_2.docx]

| Gene symbol | Full name | Coefficient |
| --- | --- | --- |
| EDN2^1^ | Endothelin 2 | 0.014 |
| CLEC3B^2^ | C-type lectin domain family 3 member B | -0.196 |
| SV2C | Synaptic vesicle glycoprotein 2C | 0.227 |
| WT1^3^ | WT1 transcription factor | 0.075 |
| MUC2^4^ | Mucin 2, oligomeric mucus/gel-forming | 0.113 |

^1^Also known as: ET-2, ET2, PPET2. ^2^Also known as: TN, TNA. ^3^Also known as: AWT1, GUD, NPHS4, WAGR, WIT-2, WT33. ^4^Also known as: MLP, MUC-2, SMUC.
