# Supplementary material for: Identification and Validation of a Five-Gene Signature Associated With Overall Survival in Breast Cancer Patients
Source: Front Oncol. 2021 Aug 26;11:660242. doi: 10.3389/fonc.2021.660242 (PMC8428534; doi:10.3389/fonc.2021.660242)
Supplement: Supplementary file 5 [file Table_3.docx]

**Supplementary Table3 Primer sets used for qPCR**

| **Primer set** | **Primers** | **Sequence(5’-3’)** |
| --- | --- | --- |
| **EDN2** | Forward | CGTCCTCATCTCATGCCCAAG |
|  | Reverse | AGGCCGTAAGGAGCTGTCT |
| **CLEC3B** | Forward | CCCAGACGAAGACCTTCCAC |
|  | Reverse | CGCAGGTACTCATACAGGGC |
| **SV2C** | Forward | TCCTACAGTCGGTTCCAAGAT |
|  | Reverse | GGCCTCACCATTATAGGTTTCTC |
| **WT1** | Forward | CACAGCACAGGGTACGAGAG |
|  | Reverse | CAAGAGTCGGGGCTACTCCA |
| **MUC2** | Forward | AGGATGACACCATCTACCTCAC |
|  | Reverse | CATCGCTCTTCTCAATGAGCA |
| **GAPDH** | Forward | GGAGCGAGATCCCTCCAAAAT |
|  | Reverse | GGCTGTTGTCATACTTCTCATGG |
